# Developing a case management program and clinical practice for high-risk sudden cardiac death patients through the lens of family systems theory

**DOI:** 10.3389/fcvm.2026.1903575

**Published:** 2026-10-14

**Authors:** Fulan Liu, Caihong Li, Zhaoguo Zhang, Jing Yang, Ke Yuan, Xuewei Jia, Wen Wang, Jiangying Luo, Dan Li, Lingling Chen, Fengli Gao, Ping Zhang

**Affiliations:** Nursing Department, Beijing Tsinghua Changgung Hospital, School of Clinical Medicine, Tsinghua Medicine, Tsinghua University, Beijing, China

**Keywords:** case management, family care, family systems theory, quality of life, sudden cardiac death

## Abstract

**Objective:**

To develop a case management plan for patients at high risk of sudden cardiac death (SCD) from the perspective of family systems theory and evaluate its clinical practice effects.

**Methods:**

By using the random sampling method, 100 patients at high risk of SCD who visited our hospital from March 2025 to June 2025 were selected as the research subjects. They were randomly divided into an experimental group (*n* = 50) and a control group (*n* = 50). The control group received routine care and follow-up, while the experimental group implemented a case management plan based on the family systems theory, with both interventions lasting for one year. Both groups were evaluated at baseline and endpoint using disease cognition assessments, the Patient Health Questionnaire-9 (PHQ-9), Generalized Anxiety Disorder-7 (GAD-7), Tampa Scale for Kinesiophobia Heart (TSK-SV Heart), General Self-Efficacy Scale (GSES), Family APGAR, and Major Adverse Cardiac Events (MACE).

**Results:**

There were no statistically significant differences in baseline data between the two groups (*P* > 0.05). After the intervention, the experimental group showed significantly better scores in disease cognition, PHQ-9, GAD-7, TSK-SV Heart, GSES, APGAR, and MACE compared to the control group (*P* < 0.05).

**Conclusion:**

The case management plan for high-risk SCD patients based on family systems theory is scientifically sound and feasible. It can effectively reduce anxiety and depression, alleviate kinesiophobia, improve disease cognition, and promote family care, thereby improving the patients’ quality of life and prognosis.

## 1 Introduction

Sudden Cardiac Death (SCD) refers to unexpected death occurring within one hour of acute symptom onset due to cardiac causes (1). Notably, SCD is a dominant contributor to global cardiovascular mortality, with estimates indicating that it accounts for 24.3%–32.25% of all cardiovascular deaths and occurs at an annual rate of 50–100 per 100,000 individuals (2–5). In China, the annual incidence of SCD reaches 544,000, ranking first globally (6). The survival rate for out-of-hospital cardiac arrest is only 10% globally, and less than 1% in China (7), imposing a tremendous psychological and economic burden on families and society (8). Studies have demonstrated that families of patients with SCD exhibit inferior levels of health literacy, unhealthy lifestyles, and insufficient access to emergency rescue resources (9). Moreover, cardiac arrest survivors and their family caregivers commonly experience anxiety, depression, and post-traumatic stress. This mutual psychological distress can adversely affect health behaviors and quality of life (10). The American Heart Association advocates for family engagement across the continuum of care as an essential strategy to enhance the quality of life in high-risk cardiovascular patients (11). The 2022 ESC Guidelines recommend familial risk screening and extended home follow-up for high-risk patients, thereby facilitating individualized prevention strategies against ventricular arrhythmias and sudden cardiac death (12). Case management provides comprehensive, continuous, and dynamic personalized services to patients, making it a critical tool for improving prognosis and quality of life (13). Globally, it has currently been applied in multiple fields including heart failure, coronary artery disease, hypertension, and cardiac rehabilitation (14–18). Bowen proposed the Family Systems Theory, which views the family as an interconnected and dynamically interacting overall system suggesting that any member's emotional changes affect and are shaped by the system (19). Michael Kerr further extended Bowen Family Systems Theory, proposing that chronic illness-related stress destabilizes the family emotional system. This systemic disturbance mediates the associations among family functioning, caregiver burden, and patient outcomes—including treatment adherence, symptom management, and long-term prognosis (20). This theoretical framework has been widely adopted to improve family dynamics and clinical outcomes among patients with diverse conditions including heart failure, breast cancer, and obesity (21–23); however, few studies have utilized it to develop interventions for individuals at high risk of sudden cardiac death and their families. Therefore, this study aims to construct a case management program for high-risk SCD patients from the perspective of family systems theory and evaluate its clinical practice effects.

## 2 Materials and methods

### 2.1 Study subjects

A total of 100 patients at high risk of sudden cardiac death, treated at the Department of Cardiovascular Medicine, Beijing Tsinghua Changgung Hospital between March 2025 and June 2025, were enrolled in this study. All eligible participants, after meeting the predefined inclusion and exclusion criteria, were assigned sequential numbers. Using a random number table generated by SPSS 23.0, participants were then randomly allocated into an intervention group and a control group, with 50 patients in each arm. The randomization sequence was securely maintained by an independent researcher who was not involved in the trial procedures. To ensure allocation concealment, group assignments were withheld from the investigators responsible for participant screening and assessments until the completion of these evaluations. According to relevant SCD guidelines and expert consensus, the inclusion criteria (24–26) were: ① Patients with genetic arrhythmias such as Brugada syndrome, Long QT Syndrome (LQTS), catecholaminergic polymorphic ventricular tachycardia, and early repolarization syndrome, accompanied by a history of cardiac arrest, malignant arrhythmia episodes, or unexplained syncope; ② Patients with cardiomyopathies such as hypertrophic cardiomyopathy, dilated cardiomyopathy, and arrhythmogenic right ventricular cardiomyopathy, accompanied by myocardial hypertrophy ≥30 mm, progressively worsening heart failure, conduction system diseases, and a history of ventricular arrhythmia; ③ Patients with coronary heart disease whose myocardial ischemia leads to a left ventricular ejection fraction (LVEF) < 40%, and who have a previous history of ventricular tachycardia episodes or cardiac arrest events; ④ Adolescents with coronary artery anatomical abnormalities, coronary artery dissection, or myocardial bridges; ⑤ Patients with a history of survival from sudden death after cardiopulmonary resuscitation (CPR). Exclusion criteria: ① Patients with comorbidities such as tumors and an expected survival of < 1 year; ② Patients already participating in other clinical studies. Withdrawal criteria: ① Subjects with poor compliance who cannot attend follow-ups on time; ② Subjects deemed unsuitable for continuing the trial by the researchers; ③ Subjects who die from any cause. The sample size was estimated based on the comparison of two sample means using the following formula:

$$n=[2\cdot (z_{\alpha }+z_{\beta }{)}^{2}\cdot \sigma^{2}]/\delta^{2}$$

Assuming a significance level *α* = 0.05 and power *β* = 0.1, the calculation resulted in n₁ = n₂ = 45. Considering a 10% loss to follow-up rate, n₁ = n₂ = 50 cases were determined. A total of 100 patients were divided into the experimental group and the control group using a random number table, with 50 cases in each group. No patients withdrew during the study process. Ultimately, 50 cases were included in the experimental group and 50 in the control group. The results of the baseline survey statistical analysis for both groups were not significant (*P* > 0.05), indicating comparability (see Table 1). This study was approved by the Hospital Ethics Committee (25041-0-01).

**Table 1:** Comparison of baseline characteristics between the two groups (xˉ±s, *n* = 100).

Variables	Categories	Control Group (*n* = 50)	Experimental Group (*n* = 50)	t or F value	*P* value
Age (years)		46.20 ± 22.56	43.08 ± 21.05	0.715	0.476
Gender	Male	34	29	1.062	0.305
	Female	16	21		
Education Level	Primary school or below	21	16	1.667	0.207
	Middle and high school	10	9		
	College and above	19	25		
Employment Status	Employed	11	24	3.705	0.067
	Retired	21	15		
	Unemployed	18	11		
Payment Type	Self-pay	8	3	0.223	0.638
	Local medical insurance	24	31		
	Non-local medical insurance	18	16		
Smoking History	Yes	18	17	0.259	0.844
	No	32	33		
Marital Status	Unmarried	13	12	0.039	0.612
	Married	32	37		
	Divorced	4	0		
	Widowed	1	1		
Number of Children	Yes	32	34	0.695	0.376
	No	18	16		
Stable Family Triangle	Yes	30	32	0.167	0.684
	No	20	18		

### 2.2 Interventions

The control group adopted a routine care and follow-up model, divided into three stages: inpatient, outpatient, and home care, for a period of 12 months. ① During hospitalization, the primary nurse provided health guidance to patients, including disease knowledge explanation, dietary guidance, medication guidance, exercise guidance, and popularization of emergency measures; ② For outpatient visits, the clinic nurse conducted health education, including disease science popularization, examination precautions, appointment procedures, medication and lifestyle guidance, and assisted in determining the time of the next visit and pre-visit preparations; ③ During the home care period, the follow-up nurse conducted regular telephone follow-ups at 1, 3, 6, and 12 months after discharge to provide health education such as medication reminders, dietary, and activity guidance, and assisted in scheduling return visits as needed. The experimental group adopted a case management model based on family systems theory.

#### 2.2.1 Establishment of the case management team

The case management team included 1 chief physician, 1 deputy chief physician, 1 attending physician, 1 head nurse, 1 ECG technician, 1 echocardiography technician, 2 case managers, and 1 professional from each of the following fields: dietitian, rehabilitation therapist, pharmacist, and social worker. Among them, the certification qualification for the case manager is a level 3 nurse who has obtained the hospital's case manager training certificate. The case manager is responsible for patient information collection, resource coordination, education and guidance, monitoring feedback, and outcome evaluation; physicians are responsible for the screening, diagnosis, treatment, and follow-up participation of patients at high risk of sudden death; technicians are responsible for patient examinations, auxiliary diagnosis, and assisting in plan formulation; the head nurse is responsible for quality control and nursing manpower coordination; social workers are responsible for the welfare resource guarantee of patients and their families.

#### 2.2.2 Theoretical basis

Family Systems Theory originated in the 1950s when Bateson's team (27) first viewed the family as an interacting system. Later, Bowen (28) and others proposed the framework of family systems theory, which is based on a stable family triangular relationship and covers core contents such as systems thinking, dependency and boundaries, and feedback and balance. Shahinbana demonstrated Family Systems Theory serves as a valuable clinical framework for identifying triangulation and emotional fusion through genograms and differentiation-of-self interventions, thereby bolstering psychological resilience in both patients and caregivers (29). The construct of differentiation of self holds substantial pragmatic value in clinical assessment and chronic disease management. Defined as the capacity to intellectually regulate emotional responses amid stressful circumstances while maintaining both personal autonomy and meaningful emotional connectedness to others (30), this construct has been empirically linked to adverse health trajectories, heightened healthcare utilization, and suboptimal treatment adherence (31). In the context of long-term care for chronic conditions such as malignancies and cardiovascular diseases, fostering differentiation of self among both patients and their caregivers has been shown to attenuate emotional reactivity and promote more deliberative, shared decision-making processes (32). Moreover, the intergenerational transmission model offers a valuable heuristic for understanding the familial propagation of health-related behaviors and emotional patterns. This framework, particularly when operationalized through genogram-based assessments, provides a robust theoretical basis for early risk identification and the design of preventive interventions targeting high-risk family lineages (33, 34).

In the 1990s, Chinese scholars (35–37) introduced family systems theory and family therapy into China, localizing the theories and techniques from the perspectives of family function, family structure, and family communication and feedback. In the field of cardiovascular diseases, studies have shown that family-centered care models are closely related to the improvement of patients' quality of life (38). For patients at high risk of sudden cardiac death, acute onset and high mortality risk pose significant challenges. Family care quality critically influences their safety and prognosis. Family systems theory-based interventions move beyond patient-centered approaches to enhance the family's caregiving system, offering a robust framework for ongoing family nursing in this high-risk population. Therefore, based on the core components of family systems theory, this study constructed a case management plan for high-risk sudden death patients encompassing three key elements: comprehensive assessment of the family system, strengthening family support and patient self-management, and outcome evaluation and feedback.

#### 2.2.3 Development of the case management plan for high-risk SCD patients based on family theory

According to the “6S” evidence pyramid model, Chinese and English databases and websites including CNKI, Wanfang Data Knowledge Service Platform, CBM, Medlive Guidelines, PubMed, UpToDate, Joanna Briggs Institute (JBI) Library, and the Cochrane Library were searched. The search period was from January 1, 2014, to March 31,2025. A “keyword + free word” search strategy was adopted. The search terms included “sudden cardiac death”, “family systems theory”, “case management”, “family-centered cardiovascular care”, “family care”, “family concern”, and “family relationship”, aiming to retrieve evidence-based case management plans for high-risk SCD patients and their families. Inclusion criteria: clear research objects; clear outcome data; articles with similar research themes. Exclusion criteria: full text unavailable; specific research type unspecified; incomplete research data; unclear research sample data; unable to contact the authors; poor literature quality evaluation. Two team members independently extracted evidence from the included literature based on the inclusion and exclusion criteria, and disagreements were resolved by a third party. The included literature was evaluated for quality using the Australian JBI Evidence-Based Health Care Centre standards. Ultimately, 7 Chinese articles (15, 21, 24, 28, 39–41) and 8 English articles (11, 38, 42–46) were included to provide an evidence-based foundation for plan formulation. A preliminary case management plan was drafted and underwent two rounds of Delphi expert consultation. The expert panel consisted of 3 cardiologists, 1 case management nursing expert, 1 cardiology nursing manager, and 1 cardiovascular specialist nurse. All panel members had 10 or more years of professional experience in this field; 5 of the 6 experts held a master's degree or above, and 1 held a bachelor's degree; 1 expert held a senior professional title, 2 held a vice-senior title, and 3 held an intermediate title. The tested Cronbach's *α* coefficient for this plan was 0.723, leading to the final formation of the case management plan for high-risk SCD patients based on family theory (see Table 2).

**Table 2:** Case management plan for high-risk SCD patients based on family systems theory.

Module	Theme	Content
1. Comprehensive Assessment of the Family System	1.1 Confirm subjects	1.1.1 Specialists screen subjects based on inclusion/exclusion criteria. 1.1.2 Case managers introduce the plan and seek consent. 1.1.3 Establish case management files for high-risk SCD patients.
	1.2 Assess conditions	1.2.1 Collect basic data, chief complaints, and diagnosis details. 1.2.2 Conduct family surveys and draw a three-generation pedigree. 1.2.3 Assess economic resources, environment, and caregiver education.
	1.3 Identify needs	1.3.1 Clarify economic, informational, and psychological needs. 1.3.2 Summarize existing problems and potential risks.
2. Strengthening Family Support & Self-Management	2.1 Formulate plan	2.1.1 Formulate inpatient diagnosis, treatment, and nursing plans. 2.1.2 Assist with financial aid applications for struggling families. 2.1.3 Jointly discuss standardized outpatient follow-up plans. 2.1.4 Train family members on home care with on-site assessments. 2.1.5 Provide risk assessments for family members with genetic risks.
	2.2 Implement intervention	2.2.1 Cognitive: Distribute manuals and explain SCD emergency measures. 2.2.2 Nutrition: Dietitians prescribe diets to control weight and lipids. 2.2.3 Exercise: Physicians formulate exercise prescriptions for rehab. 2.2.4 Medication: Continuous guidance and supervision by pharmacists. 2.2.5 Behavior: Instructions on smoking cessation and limiting alcohol. 2.2.6 Psychological: Screen for anxiety/depression; provide counselling. 2.2.7 Social: Organize community activities and seek social welfare. 2.2.8 Monitoring: Track blood pressure, heart rate, and QT interval.
3. Evaluation & Feedback	3.1 Intervention eval	3.1.1 Assess SCD cognition, family care degree, and self-efficacy. 3.1.2 Record the occurrence of major adverse cardiac events (MACE).
	3.2 Effect feedback	3.2.1 Feedback effects to the team and adjust treatment plans. 3.2.2 Provide positive guidance to promote family dynamic balance.

#### 2.2.4 Implementation of the case management plan for high-risk SCD patients based on family theory

**1. Enrollment and Assessment:** ① Establish case management files: After the specialist evaluates that the patient meets the case management inclusion criteria and obtains the consent of the patient and family members, the case manager comprehensively collects the patient's basic information, chief complaints and onset process, important examination and test results, diagnosis and treatment details, and evaluates the family relationship, living environment, physiological and psychological status, daily life habits, and the educational level of the primary caregiver. ② Family pedigree survey: The case manager investigates the composition of the patient's family members, draws a three-generation pedigree, and determines whether there is a stable triangular relationship in the patient's family (38), see Figure 1. ③ Through the evaluation results, the case manager summarizes the patient's existing problems and potential risks, clarifies the needs of the patient and family, and serves as a basis for the case management team to formulate and execute the case management plan jointly with the patient and family members.
**2. Implementation and Feedback:** During hospitalization: ① The case manager coordinates with doctors, nurses, rehabilitation therapists, dietitians, patients, and family members to formulate the most suitable diagnosis and nursing plan based on the condition and supervises its execution; ② The case management team comprehensively evaluates whether the patient meets the discharge criteria, drafts a follow-up plan, and the case manager regularly records the management plan in detail in the case management file; ③ The case manager instructs family members on home emergency knowledge and cardiopulmonary resuscitation (CPR) techniques, and conducts on-site assessments; ④ The case manager confirms the residential address, contact number, and home care situation after discharge, and social workers link community resources when necessary. Outpatient follow-up: ① According to the follow-up plan, the case manager assists the patient in scheduling return visits and informs the patient to seek emergency medical attention if uncomfortable symptoms occur; ② The case manager provides a free consultation clinic, conducts pre-diagnosis assessments for patients, and records indicator monitoring results and condition changes during the home care period; ③ Based on changes in the patient’s condition, the case manager assists the doctor in jointly optimizing the diagnosis and treatment plan, coordinates examination appointments, and tracks results; ④ The case management team provides risk assessments and free consultations for family members with genetic predispositions to prevent potential risks. Home care phase: At 1, 3, 6, and 12 months after discharge, as the patient's dedicated manager for fixed contact, the case manager uses diverse information gathering methods, including phone calls, SMS, WeChat, mini-programs, and wearable devices. Specific follow-up content includes: ① Instructing patients and families on home monitoring methods, regularly recording vital signs such as blood pressure, heart rate, and blood oxygen; ② Understanding the patient's dietary habits and ensuring balanced nutrition intake based on the advice provided by dietitians; ③ In terms of exercise, under the premise of ensuring patient safety, encouraging appropriate exercise according to suggested methods by cardiac rehabilitation therapist to enhance cardiopulmonary function and overall health; ④ Medication adherence of patients was monitored. In accordance with physicians’ orders and pharmacists’ recommendations, patients were guaranteed to take medications on time with standard dosage, and adverse drug reactions were continuously monitored.⑤ Paying attention to patient sleep quality, regulating daily routines, cultivating good hobbies, and avoiding emotional agitation to promote effective rehabilitation and management; ⑥ Instructing primary caregivers or guardians on emergency measures and equipment use in family, study, and work environments; ⑦ Detailing follow-up records and promptly reporting to relevant professional team members to ensure the patient still receives optimal nursing and medical resource support during home care. Multidisciplinary Collaboration Mechanism: A quarterly multidisciplinary team meeting was established, involving cardiologists, nurses, clinical pharmacists, dietitians, physical therapists, and social workers. During these meetings, issues identified through monthly home follow-ups were reviewed and intervention plans were modified accordingly. *Ad hoc* case conferences were convened for patients experiencing clinical deterioration. The specific responsibilities of each discipline were as follows: ① Cardiologists provided offline or online family video consultations every 3 months. Patients at high risk of syncope or implantable cardioverter-defibrillator (ICD) shocks could receive remote assessments at any time, with in-person visits arranged when clinically indicated; ② Clinical pharmacists conducted monthly medication reviews, collected medication adherence logs and adverse reaction reports, delivered personalized medication guidance, and corrected instances of missed or erroneous drug intake; ③ Clinical dietitians performed monthly nutritional assessments and formulated individualized dietary plans based on patients’ medical conditions and dietary preferences, with timely consultation available as needed; ④ Cardiac physical therapists evaluated home-based exercise tolerance via remote assessment every 3 months and adjusted rehabilitation intensity according to patients’ functional status and home activity environments; ⑤ Social workers conducted targeted interviews every 6 months for families with caregiver conflicts, patients living alone, and low-income households, and facilitated referrals to community support resources. Intervention Fidelity: To ensure fidelity and minimize performance bias, a standardized manual was developed prior to study initiation, detailing all intervention components and timelines across inpatient, outpatient, and home-based settings. All providers completed uniform training and passed competency assessments before delivering interventions. Implementation checklists were completed after each session, and 20% of case records were randomly audited monthly; protocol deviations were documented and reviewed. Fidelity was quantified by the proportion of patients completing scheduled follow-ups, monthly medication reconciliation, monthly nutritional counseling, quarterly rehabilitation guidance, and other prescribed components. This trial adopted a prospective design. The full study protocol was established before participant recruitment commenced and was rigorously adhered to throughout the entire implementation period, consistent with our pre-registered trial protocol. We further confirm that all fidelity monitoring procedures—including structured checklists, monthly supervisory audits, adherence assessment instruments, and standardized staff training protocols—were fully operationalized and executed throughout the active study phase.
3. **Exit and Evaluation:** Case management services end when the patient completes 1 year of case management, dies, refuses, or loses contact. Upon exit, the case manager evaluates the patient's symptoms, important examination indicators, disease cognition level, home management ability, and the occurrence of major adverse cardiac events.

#### 2.2.5 Effect evaluation

1. Disease cognition: At baseline and after intervention, the SCD-related disease cognition level questionnaire (47) was used to investigate disease cognition in both groups. The content includes 10 items such as SCD triggers, cardiac arrest precursors, emergency call methods, CPR knowledge, treatment methods, preventive measures, medication knowledge, dietary requirements, exercise points, and monitoring content. A 1–3 grading system is used, with a total score of 10–30 points; ≥18 indicates mastery, and <18 indicates non-mastery.
2. Psychological status: At baseline and after intervention, the Patient Health Questionnaire-9 (PHQ-9) (48), Generalized Anxiety Disorder-7 (GAD-7) (48), and The Tampa Scale for Kinesiophobia Heart (TSK-SV Heart) (49) were used to investigate the psychological status of both groups. PHQ-9 covers 9 items including loss of interest, depressed mood, sleep problems, lack of energy, appetite changes, low self-evaluation, decreased concentration, decreased or increased activity, and suicidal thoughts. A 0–3 grading system is used: 0–4 for no depression-related symptoms, 5–9 for mild depression, 10–14 for moderate depression, and 15 or above for severe depression, allowing for initial depression screening. GAD-7 includes 7 items such as feeling nervous, uncontrollable worry, excessive worry, inability to relax, difficulty concentrating, irritability, and excessive fear. A 0–3 grading system is used: 0–4 for normal level, 5–9 for mild anxiety, 10–13 for moderate anxiety, 14–18 for moderate-to-severe anxiety, and 19–21 for severe anxiety, allowing for initial anxiety screening. TSK-SV Heart includes 4 dimensions (danger perception, fear of movement, movement avoidance, and dysfunction) with 17 items. A 4-point grading system is used, with a total score of 17–68 points. Higher scores represent a more severe fear of movement, which can be used to assess kinesiophobia in heart disease patients.
3. Self-efficacy: At baseline and after intervention, the General Self-Efficacy Scale (GSES) (50) was used to evaluate self-efficacy in both groups. The scale contains 10 items covering confidence in overcoming difficulties, belief in achieving goals, emotional management in coping with stress, ability to handle complex tasks, confidence in facing challenges independently, and positive expectations for the future. A 4-point grading system is used, with a total score of 10–40 points. A higher score indicates stronger individual self-efficacy.
4. Family care degree: At baseline and after intervention, the family APGAR (Adaptation, Partnership, Growth, Affection and Resolve) scale (51) was used for evaluation. The scale contains 5 dimensions and 5 items. A 0–2 grading system is used: 7–10 indicates good family function, 4–6 indicates moderate family dysfunction, and 0–3 indicates severe family dysfunction. It is a tool for rapidly assessing family relationships.
5. Major adverse cardiac events: The occurrence frequency of Major Adverse Cardiac Events (MACE) (52) within one year of enrollment was compared between the two groups.

### 2.3 Statistical methods

#### 2.3.1 Handling of missing data

A strategy for handling missing data was pre-specified in the statistical analysis plan to address potential missing-value scenarios. Missing-data mechanisms (MCAR, MAR, MNAR) are statistical assumptions and cannot be categorized merely according to reasons for missingness. Pre-planned procedures included single imputation for secondary outcomes with <5% missingness, multiple imputation by chained equations (MICE) for primary outcomes with 5%–20% missingness, as well as pre-specified intention-to-treat (ITT) and per-protocol (PP) analyses for hypothetical premature withdrawal or loss-to-follow-up cases. Sensitivity analysis comparing complete-case and multiply-imputed datasets was also pre-planned to assess result robustness.

**Figure 1:**
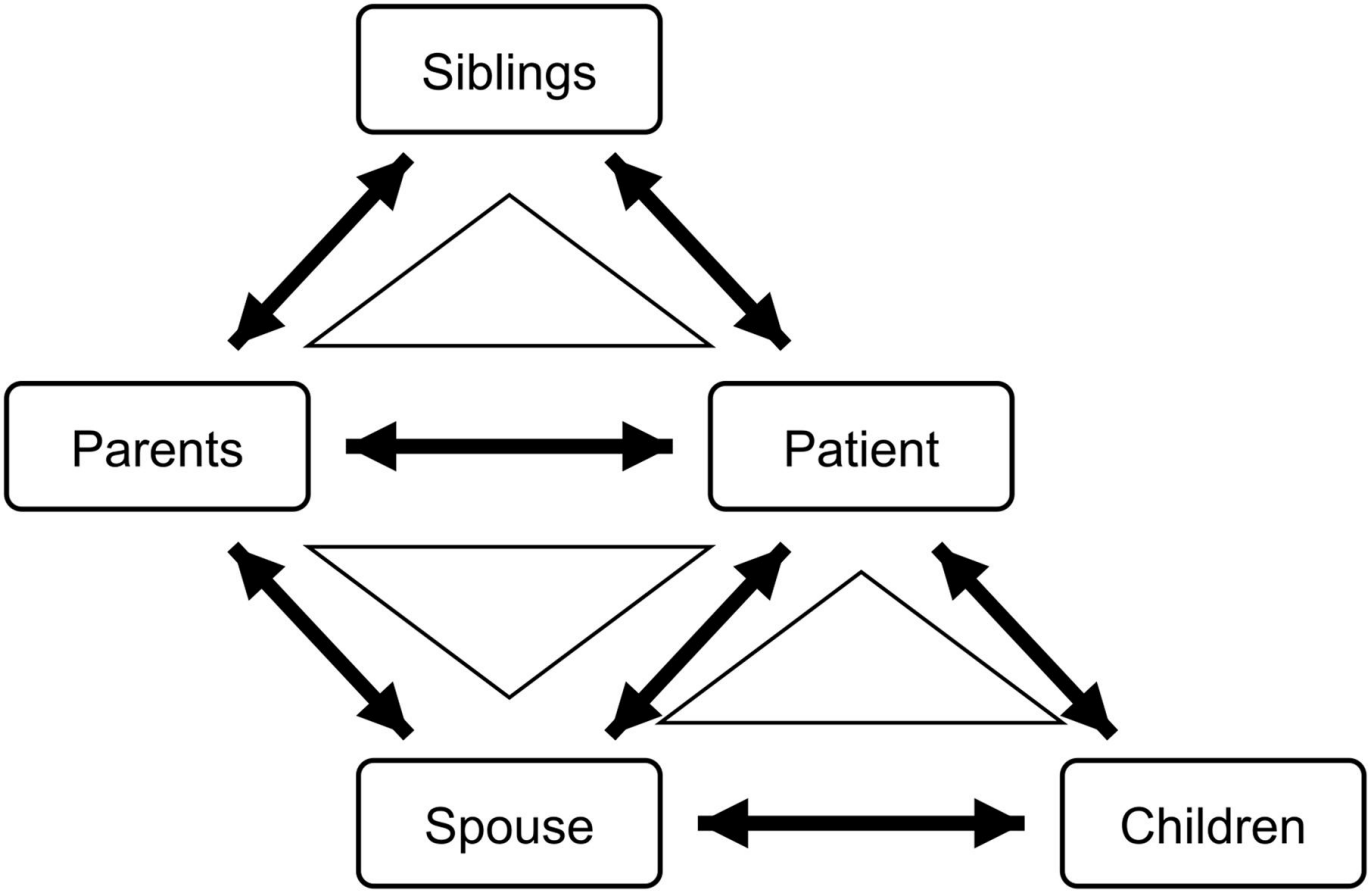
Stable family triangular relationship.

However, no participant withdrew from the study and no missing data occurred in the present trial. Therefore, none of the above-mentioned pre-specified missing-data handling procedures were applied in our actual analysis.

#### 2.3.2 Statistical analysis

Statistical analyses were performed using SPSS. The normality of continuous data was assessed using *P*-*P* plots, and all variables were found to be approximately normally distributed. Continuous variables are presented as mean ± standard deviation (SD), while categorical variables are expressed as frequencies (percentages). Baseline characteristics and post-intervention outcomes were compared between groups using independent-samples t-tests for continuous data and Pearson's chi-square tests for categorical data.

In this study, the major outcome was MACE, while disease-related cognition, PHQ-9, GAD-7, TSK-SV Heart, GSES, and APGAR were treated as exploratory secondary outcomes. The generalized estimating equation (GEE) was applied to account for within-subject correlation of repeated-measures data. The Wald test was used to evaluate the main effects and group-by-time interaction effect. Bonferroni correction for multiple comparisons was performed for the primary outcome, and a corrected *P* < 0.05 was considered statistically significant. The secondary outcomes were hypothesis-generating only and not used to draw definitive study conclusions; an uncorrected *P* < 0.05 was adopted as the threshold for statistical testing.

## 3 Results

### 3.1 Comparison of disease cognition, psychological status, self-efficacy, and family care degree between the two groups

Generalized estimating equation (GEE) revealed significant group-by-time interaction effects for disease cognition, depression (PHQ-9), anxiety (GAD-7), fear of movement (TSK-SV Heart), self-efficacy (GSES), and family care degree (APGAR) (Table 3). Participants in the experimental group demonstrated significantly greater improvements in disease cognition (*β* = 1.960, 95% CI 1.095 to 2.825, *P* < .001), self-efficacy (GSES) (*β* = 2.560, 95% CI 0.994 to 4.126, *P* = .001), and family care degree (APGAR) (*β* = 0.740, 95% CI 0.189 to 1.291, *P* = .008), as well as significantly greater reductions in depression (PHQ-9) (*β* = −1.060, 95% CI −1.630 to −0.490, *P* < .001), anxiety (GAD-7) (*β* = −2.140, 95% CI −2.983 to −1.297, *P* < .001), and fear of movement (TSK-SV Heart) (*β* = −2.560, 95% CI −4.558 to −0.562, *P* = .012) compared with those in the control group.

**Table 3:** Generalized estimating equation (GEE) analysis of repeated measures for disease cognition, psychological status, self-efficacy, and family APGAR between the two groups.

Outcome	Time point	Intervention group EM-Mean (SE) (*n* = 50)	Control group EM-Mean (SE) (*n* = 50)	Group effect *β* (95% CI)	Group effect *P*-value	Time effect β (95% CI)	Time effect *P*-value	Group × time effect β (95% CI)	Group × time effect *P*-value
SCD-Related Disease Cognition	Baseline	16.76 (0.54)	17.32 (0.53)	−0.560 (−2.049, 0.929)	0.461	NA	NA	NA	NA
	Endpoint	19.66 (0.48)	18.26 (0.44)	NA	NA	0.940 (0.431, 1.449)	＜0.001	1.960 (1.095, 2.825)	＜0.001
Depression Status (PHQ-9)	Baseline	2.24 (0.37)	2.44 (0.46)	−0.200 (−1.357, 0.957)	0.735	NA	NA	NA	NA
	Endpoint	0.98 (0.33)	2.24 (0.49)	NA	NA	−0.200 (−0.454, 0.054)	0.123	−1.060 (−1.630, −0.490)	＜0.001
Anxiety Status (GAD-7)	Baseline	3.88 (0.45)	3.12 (0.52)	0.760 (−0.587, 2.107)	0.269	NA	NA	NA	NA
	Endpoint	1.66 (0.40)	3.04 (0.53)	NA	NA	−0.080 (−0.603, 0.443)	0.764	−2.140 (−2.983, −1.297)	＜0.001
Fear of Movement (TSK-SV Heart)	Baseline	39.20 (1.10)	40.28 (1.17)	−1.080 (−4.227, 2.067)	0.501	NA	NA	NA	NA
	Endpoint	34.68 (1.15)	38.32 (1.12)	NA	NA	−1.960 (−2.944, −0.976)	＜0.001	−2.560 (−4.558, −0.562)	0.012
Self-efficacy (GSES)	Baseline	20.74 (1.46)	21.94 (1.42)	−1.200 (−5.201, 2.801)	0.557	NA	NA	NA	NA
	Endpoint	23.90 (1.54)	22.54 (1.47)	NA	NA	0.600 (−0.446, 1.646)	0.261	2.560 (0.994, 4.126)	0.001
Family Care Degree (APGAR)	Baseline	8.58 (0.23)	8.62 (0.23)	−0.040 (−0.689, 0.609)	0.904	NA	NA	NA	NA
	Endpoint	8.94 (0.18)	8.24 (0.26)	NA	NA	−0.380 (−0.712, −0.048)	0.025	0.740 (0.189, 1.291)	0.008

EM-Mean = estimated marginal mean from generalized estimating equation (GEE) with Gaussian distribution, identity link, and exchangeable correlation structure. SE, standard error; β, unstandardized coefficient; CI, confidence interval. Group effect compares intervention vs control at baseline; Time effect compares endpoint vs baseline within the control group (reference group); Group × time effect tests whether the change over time differs significantly between the two groups. NA is used for layout purposes only. Analyses were performed on all available data without imputation, as no missing data occurred.

### 3.2 Comparison of major adverse cardiac events between the two groups

Logistic generalized estimating equation analysis revealed a significant group-by-time interaction for MACE events (OR = 0.270, 95%CI 0.093 to 0.783, *P* = 0.016) (Table 4). *Post-hoc* pairwise comparisons with Bonferroni correction for multiple testing further demonstrated that MACE probability decreased significantly within the experimental group from baseline to endpoint (probability difference = −0.24, 95% CI −0.46 to −0.02, adjusted-*P* = 0.022). At endpoint, MACE probability remained significantly lower in the experimental group relative to the control group after Bonferroni correction (probability difference=−0.24, 95% CI −0.48 to −0.00, adjusted-*P* = 0.047) (Table 5). Types of major adverse cardiovascular events (MACE) are presented in Table 6.

**Table 4:** Generalized estimating equation (GEE) analysis of repeated measures for MACE events between the two groups.

Variable	OR (95% CI)	*P*-value
Group effect	1.179 (0.532, 2.610)	0.685
Time effect	1.179 (0.596, 2.332)	0.637
Group × time effect	0.270 (0.093, 0.783)	0.016

GEE with binomial distribution, logit link, and exchangeable correlation structure. OR, odds ratio; CI, confidence interval. The Group × Time interaction tests whether the change in MACE risk from baseline to endpoint differs significantly between the two groups.

**Table 5:** *Post-hoc* pairwise comparisons (probability difference scale).

Comparison	Difference in probability (95% CI)	Adjusted-*P*
Intervention group: Baseline vs Endpoint	−0.24 (−0.46, −0.02)	0.022
Endpoint: Intervention vs Control	−0.24 (−0.48, 0.00)	0.047
Baseline: Intervention vs Control	0.04 (−0.22, 0.30)	>0.999
Control group: Baseline vs Endpoint	0.00 (−0.26, 0.26)	>0.999

Pairwise comparisons were performed on the original probability scale with Bonferroni correction, following a significant group-by-time interaction in the logistic generalized estimating equation model.

**Table 6:** Types of MACE in the two groups.

Group	Control		Experimental	
	Baseline	Endpoint	Baseline	Endpoint
Unplanned readmission for cardiovascular causes	2	3	2	1
Presyncope	5	8	4	5
Syncope	10	6	12	3
Malignant arrhythmia	2	3	2	1
appropriate ICD shock	0	1	1	0
Cardiac arrest	1	0	1	0
Total	20	21	22	10

## 4 Discussion

### 4.1 Case management for high-risk SCD patients from the perspective of family systems theory can improve disease cognition and psychological status

This study showed that after intervention, the PHQ-9, GAD-7, and TSK-SV Heart scores of the experimental group were lower than those of the control group, with statistical significance (*P* < 0.05), indicating that the case management plan centered around the affected family effectively alleviated the patients' depression, anxiety, and kinesiophobia. Following intervention, the experimental group demonstrated significantly higher scores in SCD-related disease cognition and the General Self-Efficacy Scale (GSES) relative to the control group (*P* < 0.05), reflecting superior disease cognition and self-efficacy and verifying that this intervention can effectively enhance patients' disease cognition and self-management capacity. In addition, the experimental group showed a larger mean increase in GSES scores, indicating a potential dose-response relationship between intervention duration and improvements in self-efficacy; therefore, longer-term follow-up may be needed to guarantee sustained benefits (53). This is similar to the findings of Han Meng (54), where a full-course health education model based on the family unit can improve patients' psychological status and effectively enhance self-efficacy. This result benefits from the case management team's full-course care, spanning hospitalization, outpatient follow-up, and home care, which facilitates the timely discovery and treatment of changes in the patient's condition, strengthens health education, and comforts patients' fears, reflecting the potential value of this case management plan in reducing kinesiophobia and enhancing patients' confidence and coping abilities.

### 4.2 Case management for high-risk SCD patients from the perspective of family systems theory can promote family care

Research shows that the family care score of the experimental group was significantly higher than before intervention, while the control group showed a downward trend in family care scores (*P* < 0.05). This indicates that family system intervention not only improves the patient's mental health but also promotes care and support among family members. Multiple studies show (55, 56) that family caregivers play a crucial role in delaying disease progression and improving the quality of life for cardiovascular patients. Therefore, a good family support environment helps enhance the patient's sense of emotional security and belonging, making them feel less isolated when facing illness and more actively engaged in their rehabilitation. This phenomenon of increased family care reflects the important role played by the case management team in promoting family function and strengthening family bonds.

### 4.3 Case management for high-risk SCD patients from the perspective of family systems theory can improve prognosis

Within one year of intervention, the incidence of major adverse cardiac events in the experimental group was significantly lower than in the control group, and the difference was statistically significant (P＜0.05). This finding not only demonstrates the positive impact of improved mental health and enhanced family support on physical health but also confirms the promoting role of the family-perspective case management plan in reducing the risk of adverse events in high-risk SCD patients. This can be explained by the fact that negative emotional interactions within families sustain elevated sympathetic activation and provoke repolarization abnormalities. Long-term intervention targeting the entire family unit is therefore required, whereas isolated short-term in-hospital intervention lacks long-term preventive effects against sudden cardiac death (57). This is consistent with the findings of Jiaoyu Cao (58), where family-based intervention models have a positive impact on the long-term effects of cardiovascular patients. Grounded in family systems theory, family-based interventions not only increase screening uptake among first-degree relatives but also mitigate the familial SCD burden, suggesting that such approaches confer prognostic benefits for at-risk individuals (59). This result provides strong empirical support for introducing family systems theory into cardiovascular disease management in the future and emphasizes the importance of case management in elevating nursing quality and improving prognosis.

## 5 Conclusion

This study constructed a case management plan for high-risk sudden cardiac death patients based on family systems theory and evaluated its clinical practice effects. The results show that it can effectively reduce adverse emotions such as anxiety, depression, and kinesiophobia, improve disease cognition levels, and promote family members’ care, thereby improving patients' quality of life and prognosis. In addition, due to economic constraints and limited funding, only partial genetic-risk assessments for at-risk family members were completed, which could attenuate the full potential efficacy of this family-centered intervention. It is hoped that future case management teams can link with community hospitals and other grassroots medical forces to complete broader screenings of high-risk populations, preventing more sudden cardiac death events from the source.

## Data Availability

The original contributions presented in the study are included in the article/Supplementary Material, further inquiries can be directed to the corresponding authors.
