# Supplementary material for: Developing a case management program and clinical practice for high-risk sudden cardiac death patients through the lens of family systems theory
Source: Front Cardiovasc Med. 2026 Oct 14;13:1903575. doi: 10.3389/fcvm.2026.1903575 (PMC13713636; doi:10.3389/fcvm.2026.1903575)
Supplement: Supplementary file 1 [file Table1.doc]

****Table 1.** Comparison of Baseline Characteristics Between the Two Groups**

**(xˉ±s, n=100)**

| **Variables** | **Categories** | **Control Group (n=50)** | **Experimental Group (n=50)** | **t or F value** | **P value** |
| --- | --- | --- | --- | --- | --- |
| **Age (years)** |  | 46.20±22.56 | 43.08±21.05 | 0.715 | 0.476 |
| **Gender** | Male | 34 | 29 | 1.062 | 0.305 |
|  | Female | 16 | 21 |  |  |
| **Education Level** | Primary school or below | 21 | 16 | 1.667 | 0.207 |
|  | Middle and high school | 10 | 9 |  |  |
|  | College and above | 19 | 25 |  |  |
| **Employment Status** | Employed | 11 | 24 | 3.705 | 0.067 |
|  | Retired | 21 | 15 |  |  |
|  | Unemployed | 18 | 11 |  |  |
| **Payment Type** | Self-pay | 8 | 3 | 0.223 | 0.638 |
|  | Local medical insurance | 24 | 31 |  |  |
|  | Non-local medical insurance | 18 | 16 |  |  |
| **Smoking History** | Yes | 18 | 17 | 0.259 | 0.844 |
|  | No | 32 | 33 |  |  |
| **Marital Status** | Unmarried | 13 | 12 | 0.039 | 0.612 |
|  | Married | 32 | 37 |  |  |
|  | Divorced | 4 | 0 |  |  |
|  | Widowed | 1 | 1 |  |  |
| **Number of Children** | Yes | 32 | 34 | 0.695 | 0.376 |
|  | No | 18 | 16 |  |  |
| **Stable Family Triangle** | Yes | 30 | 32 | 0.167 | 0.684 |
|  | No | 20 | 18 |  |  |
