# Supplementary material for: Developing a case management program and clinical practice for high-risk sudden cardiac death patients through the lens of family systems theory
Source: Front Cardiovasc Med. 2026 Oct 14;13:1903575. doi: 10.3389/fcvm.2026.1903575 (PMC13713636; doi:10.3389/fcvm.2026.1903575)
Supplement: Supplementary file 2 [file Table2.doc]

****Table 2.** Case Management Plan for High-Risk SCD Patients Based on Family Systems Theory**

| **Module** | **Theme** | **Content** |
| --- | --- | --- |
| **1. Comprehensive Assessment of the Family System** | 1.1 Confirm subjects | 1.1.1 Specialists screen subjects based on inclusion/exclusion criteria.  1.1.2 Case managers introduce the plan and seek consent.  1.1.3 Establish case management files for high-risk SCD patients. |
|  | 1.2 Assess conditions | 1.2.1 Collect basic data, chief complaints, and diagnosis details.  1.2.2 Conduct family surveys and draw a three-generation pedigree.  1.2.3 Assess economic resources, environment, and caregiver education. |
|  | 1.3 Identify needs | 1.3.1 Clarify economic, informational, and psychological needs.  1.3.2 Summarize existing problems and potential risks. |
| **2. Strengthening Family Support & Self-Management** | 2.1 Formulate plan | 2.1.1 Formulate inpatient diagnosis, treatment, and nursing plans.  2.1.2 Assist with financial aid applications for struggling families.  2.1.3 Jointly discuss standardized outpatient follow-up plans.  2.1.4 Train family members on home care with on-site assessments.  2.1.5 Provide risk assessments for family members with genetic risks. |
|  | 2.2 Implement intervention | 2.2.1 Cognitive: Distribute manuals and explain SCD emergency measures.  2.2.2 Nutrition: Dietitians prescribe diets to control weight and lipids.  2.2.3 Exercise: Physicians formulate exercise prescriptions for rehab.  2.2.4 Medication: Continuous guidance and supervision by pharmacists.  2.2.5 Behavior: Instructions on smoking cessation and limiting alcohol.  2.2.6 Psychological: Screen for anxiety/depression; provide counselling.  2.2.7 Social: Organize community activities and seek social welfare.  2.2.8 Monitoring: Track blood pressure, heart rate, and QT interval. |
| **3. Evaluation & Feedback** | 3.1 Intervention eval | 3.1.1 Assess SCD cognition, family care degree, and self-efficacy.  3.1.2 Record the occurrence of major adverse cardiac events (MACE). |
|  | 3.2 Effect feedback | 3.2.1 Feedback effects to the team and adjust treatment plans.  3.2.2 Provide positive guidance to promote family dynamic balance. |
