# Supplementary material for: Developing a case management program and clinical practice for high-risk sudden cardiac death patients through the lens of family systems theory
Source: Front Cardiovasc Med. 2026 Oct 14;13:1903575. doi: 10.3389/fcvm.2026.1903575 (PMC13713636; doi:10.3389/fcvm.2026.1903575)
Supplement: Supplementary file 3 [file Table3.doc]

****Table 3.** Generalized estimating equation (GEE) analysis of repeated measures for disease cognition, psychological status, self-efficacy, and family APGAR between the two groups.**

| Outcome | time point | Intervention group EM-Mean (SE) (n=50) | Control group  EM-Mean (SE) (n=50) | Group effectβ (95% CI) | Group effect P-value | Time effectβ (95% CI) | Time effect P-value | Group×time effectβ (95% CI) | Group×time effect P-value |
| --- | --- | --- | --- | --- | --- | --- | --- | --- | --- |
| **SCD-Related Disease Cognition** | Baseline | 16.76 (0.54) | 17.32 (0.53) | −0.560 (−2.049, 0.929) | 0.461 | NA | NA | NA | NA |
|  | Endpoint | 19.66 (0.48) | 18.26 (0.44) | NA | NA | 0.940 (0.431, 1.449) | ＜0.001 | 1.960 (1.095, 2.825) | ＜0.001 |
| **Depression Status (PHQ-9)** | Baseline | 2.24 (0.37) | 2.44 (0.46) | −0.200 (−1.357, 0.957) | 0.735 | NA | NA | NA | NA |
|  | Endpoint | 0.98 (0.33) | 2.24 (0.49) | NA | NA | −0.200 (−0.454, 0.054) | 0.123 | −1.060 (−1.630, −0.490) | ＜0.001 |
| **Anxiety Status (GAD-7)** | Baseline | 3.88 (0.45) | 3.12 (0.52) | 0.760 (−0.587, 2.107) | 0.269 | NA | NA | NA | NA |
|  | Endpoint | 1.66 (0.40) | 3.04 (0.53) | NA | NA | −0.080 (−0.603, 0.443) | 0.764 | −2.140 (−2.983, −1.297) | ＜0.001 |
| **Fear of Movement (TSK-SV Heart)** | Baseline | 39.20 (1.10) | 40.28 (1.17) | −1.080 (−4.227, 2.067) | 0.501 | NA | NA | NA | NA |
|  | Endpoint | 34.68 (1.15) | 38.32 (1.12) | NA | NA | −1.960 (−2.944, −0.976) | ＜0.001 | −2.560 (−4.558, −0.562) | 0.012 |
| **Self-efficacy（GSES）** | Baseline | 20.74 (1.46) | 21.94 (1.42) | −1.200 (−5.201, 2.801) | 0.557 | NA | NA | NA | NA |
|  | Endpoint | 23.90 (1.54) | 22.54 (1.47) | NA | NA | 0.600 (−0.446, 1.646) | 0.261 | 2.560 (0.994, 4.126) | 0.001 |
| **Family Care Degree（APGAR）** | Baseline | 8.58 (0.23) | 8.62 (0.23) | −0.040 (−0.689, 0.609) | 0.904 | NA | NA | NA | NA |
|  | Endpoint | 8.94 (0.18) | 8.24 (0.26) | NA | NA | −0.380 (−0.712, −0.048) | 0.025 | 0.740 (0.189, 1.291) | 0.008 |

Footnote: EM-Mean=estimated marginal mean from generalized estimating equation (GEE) with Gaussian distribution, identity link, and exchangeable correlation structure. SE=standard error; β=unstandardized coefficient; CI=confidence interval. Group effect compares intervention vs control at baseline; Time effect compares endpoint vs baseline within the control group (reference group); Group×time effect tests whether the change over time differs significantly between the two groups. NA is used for layout purposes only. Analyses were performed on all available data without imputation, as no missing data occurred.
